# lncRNA-AC130710/miR-129-5p/mGluR1 axis promote migration and invasion by activating PKCα-MAPK signal pathway in melanoma

**DOI:** 10.1515/med-2022-0587

**Published:** 2022-10-18

**Authors:** Zhi Xie, Chen Wang, Li Li, Xianfeng Chen, Guanjing Wei, Yan Chi, Yanping Liang, Lizhen Lan, Jiqiong Hong, Lili Li

**Affiliations:** Department of Dermatology, People’s Hospital of Guangxi Zhuang Autonomous Region, Nanning 530021, PR China; Department of Intensive Care Unit, The First Affiliated Hospital of Guangxi Medical University, Nanning 530021, PR China; Department of Dermatology, People’s Hospital of Guangxi Zhuang Autonomous Region, 6 Taoyuan Road, Nanning 530021, PR China

**Keywords:** melanoma, lncRNA, mGluR1, migration, invasion

## Abstract

Invasion and metastasis of melanoma are a series of complicated biological events regulated by multiple factors. The coregulation of many molecules involved in the development and progression of melanoma contributes to invasion and migration. mGluR1 is a metabotropic glutamate receptor that is overexpressed in melanocytes and is sufficient to induce melanoma. In our study, we found that mGluR1 was obviously increased in melanoma. Furthermore, we found that miR-129-5p could directly target and regulate mGluR1 mRNA, which was significantly reduced in A375 cells. Overexpression of miR-129-5p inhibited cell migration, invasion and clonal formation. lncRNA-AC130710 directly targeted and suppressed miR-129-5p in A375 cells. Downregulation of lncRNA-AC130710 suppressed the levels of mGluR1 mRNA by promoting miR-129-5p expression and further inhibiting migration, invasion and colony formation in A375 cells, which was associated with the activation of the PKCα-MAPK signaling pathway. Taken together, our study showed that the lncRNA-AC130710/miR-129-5p/mGluR1 axis plays an important role in the invasion and metastasis of melanoma.

## 1 Introduction

Malignant melanoma is a type of malignant tumor that originates from neural crest melanoma cells [1]. Although it accounts for only approximately 4% of all dermatological cancers, it contributes to more than 80% of deaths in skin cancer patients [2]. The invasion and metastasis of melanoma is a complex process with multiple stages and is affected by many factors that directly affect the melanoma prognosis for patients [3,4]. The coregulation of many molecules contributes to invasion and migration, which thereby participates in the development and progression of melanoma.

Metabotropic glutamate receptors (mGluRs) can mediate neuronal excitability and neurotransmitter release and have been extensively studied in the central nervous system. Certain cancers, such as melanoma, express various mGluR subtypes that might play a role in disease progression. Namkoong et al. and Lee et al. found abnormal expression of mGluR1 in human melanoma cell lines and tissue sections [5,6]. Suppression of mGluR1 and glutamate signaling can inhibit the progression of melanoma [5]. In our previous study, we also found that mGluR1 expression was elevated in melanoma. Therefore, we wanted to inhibit the invasion and metastasis of melanoma by regulating mGluR1.

lncRNAs are noncoding RNAs of the more than 200 nt in length and regulate various biological events [7–9]. Recently, studies have shown that there are multiple interactions between noncoding RNAs and coding RNAs, such as the lncRNA‒miRNA–mRNA regulatory network [10]. As a representative mechanism, lncRNAs can function as miRNA sponges [11]. For example, Wei et al. demonstrated that the lncRNA UCA1-miR-507-FOXM1 axis participates in melanoma cell proliferation and invasion by regulating the G_0_/G_1_ cell cycle [12]. Sun et al. showed that lncRNA MALAT1, as the competitive endogenous RNA sponge of miR-183, promoted the occurrence and development of melanoma by regulating the miR-183-ITGB1 axis [13]. Therefore, elucidating the regulatory network of lncRNA–miRNA–mRNA is critical to determine the specific role of each molecule in the metastasis and invasion of malignant melanoma [14,15]. Xu et al. determined that lncRNA-AC130710 plays an important role in regulating the invasion of gastric cancer cells by targeting miR-129-5p [16]. Previously, we predicted the interaction between miR-129-5p and mGluR1 by miRanda (www.microrna.org) and RNAhybrid (https://bibiserv.cebitec.uni-bielefeld.de/rnahybrid/).

Therefore, we wanted to explore whether lncRNA-AC130710 promoted melanoma invasion and metastasis by interacting with miR-129-5p to upregulate the expression of mGluR1.

## 2 Materials and methods

### 2.1 Cell culture

A375 cells, HEK-293T cells and a human primary melanocyte cell line were purchased from iCell Bioscience Inc. (Shanghai, China). A375 cells were cultured in DMEM with 10% fetal bovine serum (FBS, HyClone) and penicillin‒streptomycin (100 U/mL) at 37°C with 5% CO_2_. The human primary melanocyte cell line was cultured in a special medium for primary melanocytes (iCell Bioscience Inc.) with 0.5% FBS (HyClone), special culture additives for primary melanocyte cell lines (iCell Bioscience Inc.) and penicillin‒streptomycin (100 U/mL) at 37°C with 5% CO_2_. HEK-293T cells were cultured in DMEM with 10% FBS, 1% Glutamax, 1% NEAA and penicillin‒streptomycin (100 U/mL) at 37°C with 5% CO_2_.

### 2.2 Cell transfection

The miR-129-5p mimic, negative control (NC) mimic, three lncRNA-AC130710 siRNAs and one siRNA NC were synthesized by Sangon Biotech (Shanghai, China). The sequences were as follows: hsa-miR-129-5p-S, CUUUUUGCGGUCUGGGCUUGC; hsa-miR-129-5p-A, AAGCCCAGACCGCAAAAAGUU; mimic NC-S, UUGUACUACACAAAAGUACUG and mimic NC-A, GUACUUUUGUGUAGUACAAUU. The vectors used in our study included pcDNA3.1-lncRNA-AC130710 and pcDNA3.1 NC. A375 cells were transfected using Lipofectamine 2000 (Invitrogen, USA) for 24 h according to the manufacturer’s instructions.

### 2.3 Real-time qPCR

Total RNA was extracted from cells using the TaKaRa MiniBEST Universal RNA Extraction Kit according to the manufacturer’s procedure. Then, cDNA was synthesized by using a miRNA 1st Strand cDNA Synthesis Kit (Vazyme) and Hifair^®^Ⅱ 1st Strand cDNA Synthesis SuperMix (YEASEN, Shanghai). After that, Hieff^®^ qPCR SYBR Green Master Mix (YEASEN, Shanghai) was used to perform the RT-qPCR on an ABI QuantStudio™ 12K Flex according to the manufacturer’s procedure. The qPCR conditions were as follows: 95°C for 5 min followed by 40 cycles of 95°C for 10 s, 55–60°C for 20 s and 72°C for 20 s. The primers used were as follows: mGluR1, F: 5′–CCAACTTCAACGAGGCCAAA–3′, R: 5′–CATGCGGACAACATCAGAGG–3′; miR-129-5p, F: 5′–CGCTTTTTGCGGTCTGG–3′, R: 5′–CAGTGCGTGTCGTGGAGT–3′; lncRNA-AC130710: F: 5′–AGGACAGTCTCAAGGGGGTTA–3′, R: 5′–CTGCCTTCTCACATGGAACTC–3′; GAPDH: F, 5′–TCAAGAAGGTGGTGAAGCAGG–3′, R: 5′–TCAAAGGTGGAGGAGTGGGT–3′ and U6, F: 5′–GCTTCGGCAGCACATATACTAAAAT–3′, R: 5′–CGCTTCACGAATTTGCGTGTCAT–3′. The primers were obtained from Sangon Biotech (Shanghai, China). The levels of mGluR1 and lncRNA-AC130710 were normalized to that of GAPDH. The levels of miR-129-5p were normalized to that of U6. The relative expression levels of genes were calculated using the 2^−ΔΔCt^ method.

### 2.4 Luciferase reporter assay

mGluR1-WT-pmirGLO, mGluR1-Mut-pmirGLO, lncRNA-AC130710-WT-pmirGLO and lncRNA-AC130710-Mut-pmirGLO plasmids were constructed by GenScript (Wuhan, China). Cells were seeded in 24-well plates and grown for 24 h before transfection. Lipofectamine 2000 (Invitrogen, USA) was used to cotransfect miR-129-5p mimics or the miR-NC and wild-type or mutant plasmids into cells. Transfected cells were harvested after 48 h and then analyzed using a Dual-Luciferase Reporter Assay System (Promega, USA).

### 2.5 Wound healing assay

A375 cells were transfected with miR-129-5p mimic, mimic NC, pcDNA3.1-lncRNA-AC130710, pcDNA3.1 NC, lncRNA-AC130710 siRNA or siRNA NC for 48 h, respectively. Then, these cells were seeded into six-well plates at a density of 3.5 × 10^5^ cells per well at 37°C. A 10 μL pipette tip was used to make a straight scratch. The culture medium was discarded, and the cells were washed three times in PBS. Then, serum-free medium was added. An Olympus IX71 microscope was used to take images at 0, 24 and 48 h after scratching, and then the migration distances of the cells were calculated. Scratch width was subtracted from time 0 scratch widths at the same location to determine cell migration distance. Migration distances were averaged to determine overall migration.

### 2.6 Invasion assays

Cell invasive capacity was assessed by using a transwell chamber. Matrigel was diluted ten times with serum-free medium, and 50 µL was added to each transwell chamber, followed by incubation at 37°C for 30 min. Transfected cells were harvested and suspended in serum-free DMEM. Then, 300 μL of cell suspension (2 × 10^4^/mL cells) was placed into the upper chamber, and 600 μL medium containing 10% FBS was added to the lower chamber. Cells were cultured at 37°C for 24 h. After 24 h, cells on the upper surface were removed with a cotton swab, while the invasive cells in the lower chamber were fixed with 4% paraformaldehyde and stained with crystal violet. The stained cells were counted in three randomly selected visual fields per well under an Olympus IX71 microscope.

### 2.7 Colony formation assay

Transfected cells were seeded into six-well plates at a density of 200 cells/well and cultured at 5% CO_2_ and 37°C for 2 weeks. Cells were washed with PBS, fixed with 4% paraformaldehyde for 10 min and stained with crystal violet. The colony number was then determined with an Olympus IX71 microscope. The assays were independently repeated three times.

### 2.8 Western blot analyses

Total cellular protein was quantified using a BCA protein assay kit (Thermo Fisher Scientific). Equal amounts of protein were separated by 12% SDS‒PAGE and transferred to a polyvinylidene fluoride membrane (Hybond, CA). The membranes were blocked and then incubated overnight at 4°C with antibodies against mGluR1, PKCα, MAPK42/44, p-MAPK42/44 and GAPDH (1:1,000, ABclonal). The membranes were incubated with secondary antibodies conjugated to horseradish peroxidase (1:5,000; Beyotime Biotechnology, China) at room temperature for 2 h. Protein bands were detected using an efficient enhanced chemiluminescence (ECL) kit (GE Healthcare, UK) and quantitated using Quantity One software (Bio-Rad Laboratories, UK). Band intensities were normalized to that of GAPDH.

### 2.9 Statistical analysis

All data are expressed as the mean ± standard deviation. Statistical analysis was carried out using SPSS 13.0. Comparisons among multiple groups were conducted using one-way analysis of variance; *p* < 0.05 was considered to indicate a statistically significant difference.

## 3 Results

### 3.1 Upregulated miR-129-5p suppresses mGluR1 expression in melanoma

**Figure 1:**
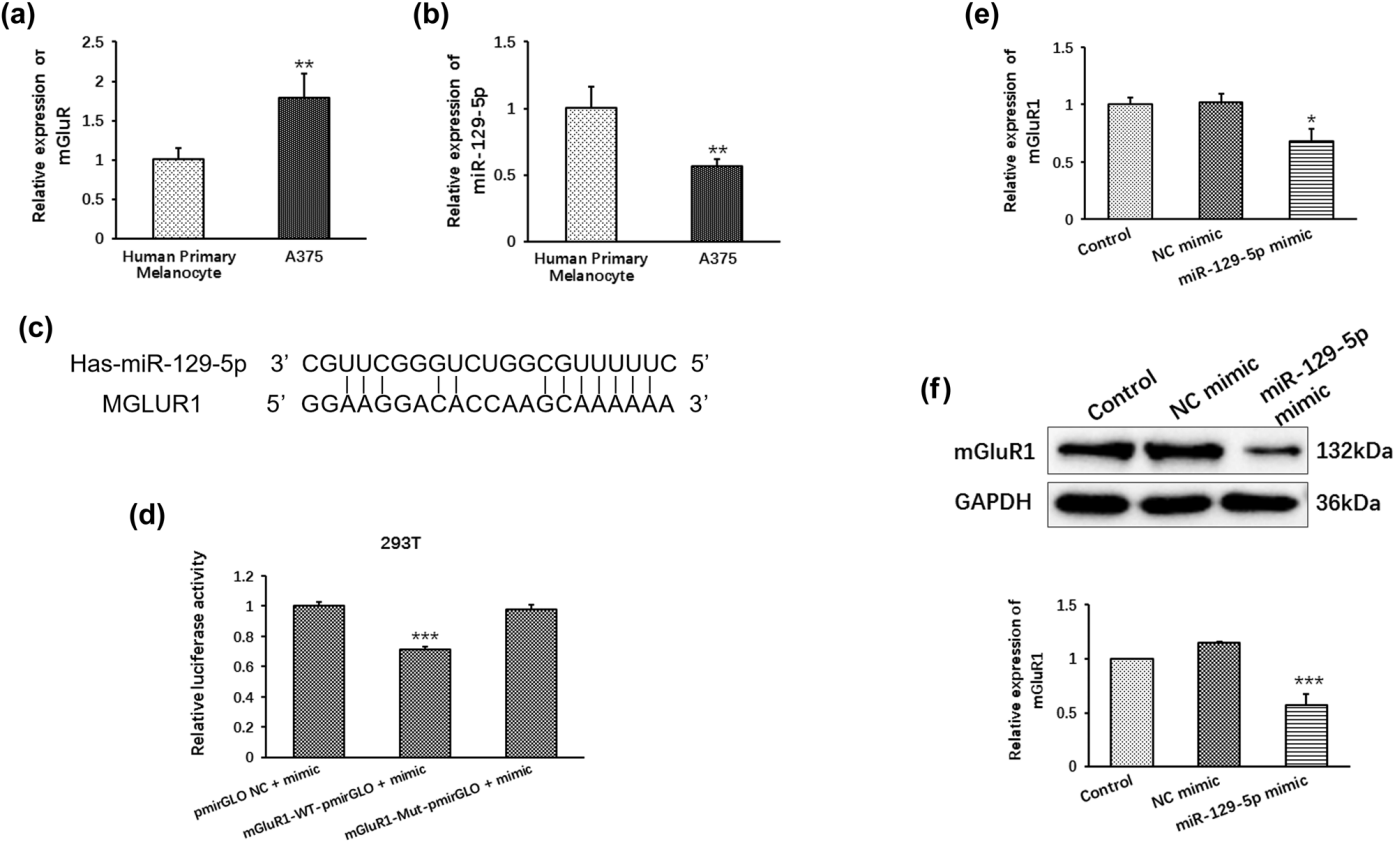
miR-129-5p directly target with mGluR1 in A375. (a) Relative mRNA expression of mGluR1 in A375 cells compared with human primary melanocytes cell line. (b) Relative mRNA expression of miR-129-5p in A375 cells compared with human primary melanocytes cell line. (c) Predicted complementary binding site of miR-129-5p in mGluR1 3′-UTR. (d) Relative luciferase activities were inhibited transfected with the reporter vector WT mGluR1, not with the reporter vector Mut mGluR1. (e) Relative mRNA expression of mGluR1 detected by qPCR in A375 cells were inhibited transfected with miR-129-5p mimic. (f) Relative expression of mGluR1 detected by Western blot in A375 cells were inhibited transfected with miR-129-5p mimic. Data are presented as means ± SD (*n* = 3; *p < 0.05, **p < 0.01, ***p < 0.001).

mGluR1 is a metabotropic glutamate receptor, and mGluR1 expression in mouse melanocytes was determined to be sufficient to induce melanoma. Consistent with a previous study, our results showed that the expression of mGluR1 was significantly increased in A375 cells compared with the human primary melanocyte cell line (Figure 1a).

Our previous study found that the level of miR-129-5p was obviously decreased in A375 cells compared with the human primary melanocyte cell line (Figure 1b), and we also found a targeting relationship between miR-129-5p and mGluR1. We used miRanda (www.microrna.org) and RNAhybrid (https://bibiserv.cebitec.uni-bielefeld.de/rnahybrid/) to predict the putative complementary binding sites of miR-129-5p and mGluR1 (Figure 1c).

Furthermore, we wanted to determine whether miR-129-5p directly targets mGluR1 using a luciferase reporter assay. As shown in Figure 1d, overexpression of miR-129-5p decreased the luciferase activity of the wild-type mGluR1 3′-UTR reporter in 293T cells. However, miR-129-5p mimics did not influence the luciferase activity of the reporter carrying the mutated mGluR1 3′-UTR.

Moreover, the addition of miR-129-5p resulted in significantly downregulated expression of mGluR1 in both the qPCR and Western blot results (Figure 1e and f).

### 3.2 Overexpression of miR-129-5p affects cell migration, cell invasion and colony formation in melanoma

**Figure 2:**
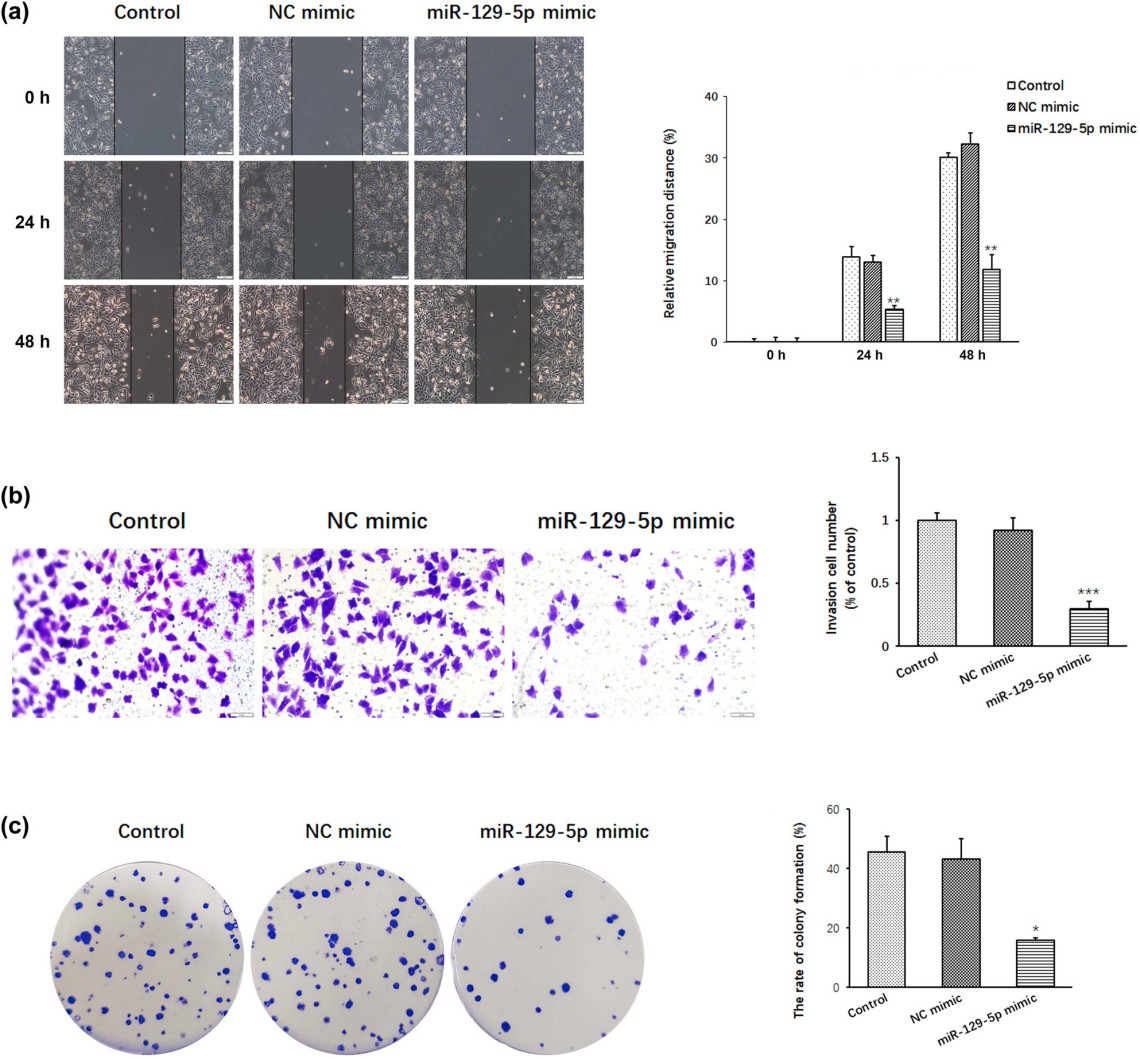
Effects of miR-129-5p overexpression on the migration, invasion and clonal formation of A375 cells. (a) Cell migration was investigated by wound healing scratch assay in A375 cells transfected with miR-129-5p. (b) Cell invasion was investigated in A375 cells transfected with miR-129-5p. (c) Colony formation of A375 cells transfected with miR-129-5p. Data are presented as means ± SD (*n* = 3; *p < 0.05, **p < 0.01, ***p < 0.001).

We examined the effect of miR-129-5p on cell migration, cell invasion and colony formation in A375 cells (Figure 2). The results of the scratch assay showed that the relative migration distance was 5.32% at 24 h and 11.82% at 48 h in cells transfected with the miR-129-5p mimic, and the relative migration distance was 13.9% at 24 h and 30.11% at 48 h in control cells (Figure 2a). Overexpression of miR-129-5p significantly inhibited A375 cell migration by 61.72% at 24 h and 60.74% at 48 h compared with the control.

We also examined the effect of miR-129-5p expression on the invasion of A375 cells (Figure 2b). The results of the invasion assay showed that overexpressed miR-129-5p inhibited A375 cell invasion by 70.29%, which showed obvious inhibition of melanoma cell invasion.

The effect of miR-129-5p on the proliferation capacity of tumor cells was also examined with the colony formation assay. When plated at a density of 200 cells/well, miR-129-5p mimic-transfected cells generated a lower rate of colony formation (15.83%) than control cells (45.67%) (Figure 2c). Overexpression of miR-129-5p significantly inhibited cell migration, cell invasion and colony formation in melanoma.

### 3.3 lncRNA-AC130710 increases in melanoma and inhibits miR-129-5p expression

**Figure 3:**
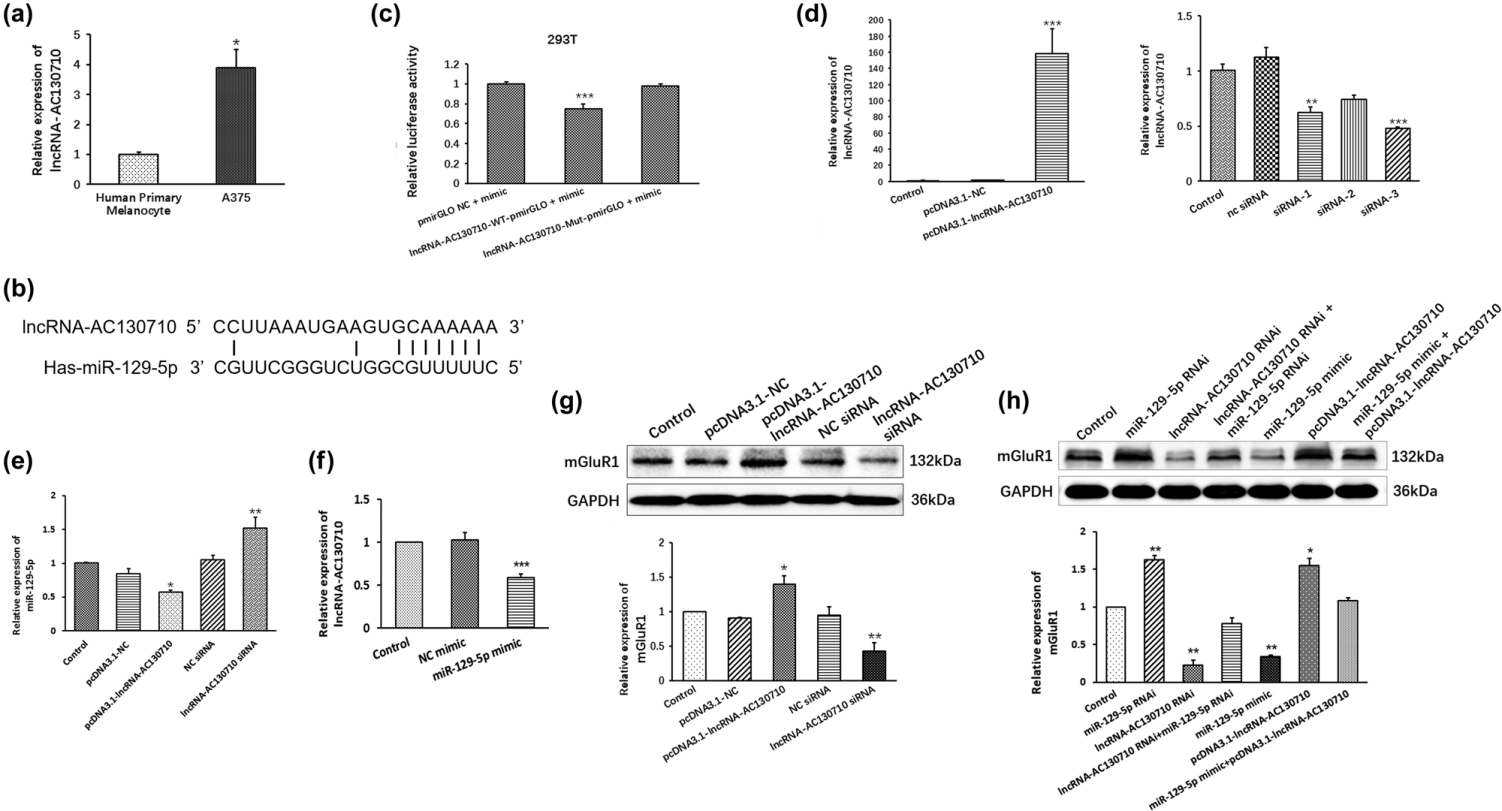
lncRNA-AC130710 directly target with miR-129-5p in A375. (a) Relative expression of lncRNA-AC130710 in A375 cells compared with human primary melanocytes cell line. (b) Predicted complementary binding site of miR-129-5p in lncRNA-AC130710. (c) Relative luciferase activities were inhibited transfected with the reporter vector WT lncRNA-AC130710, not with the reporter vector Mut lncRNA-AC130710. (d) Construction of pcDNA3.1-lncRNA-AC130710 and lncRNA-AC130710 siRNA vectors. (e) Relative expression of miR-129-5p in A375 cells transfected with pcDNA3.1-lncRNA-AC130710 and lncRNA-AC130710 siRNA vectors. (f) Relative expression of lncRNA-AC130710 in A375 cells transfected with NC mimic and miR-129-5p mimic vectors. (g) Relative expression of mGluR1 in A375 cells transfected with pcDNA3.1-lncRNA-AC130710 and lncRNA-AC130710 siRNA vectors. (h) Relative expression of mGluR1 in A375 cells when modulation of miR-129-5p or lncRNA-AC130710 expression. Data are presented as means ± SD (*n* = 3; *p < 0.05, **p < 0.01, ***p < 0.001).

Previous research has determined that lncRNA-AC130710 directly targets and suppresses miR-129-5p in gastric cancer. Here, we wanted to verify whether there is a relationship between lncRNA-AC130710 and miR-129-5p in melanoma. First, we determined the level of lncRNA-AC130710 in A375 cells and the human primary melanocyte cell line. We found that the level of lncRNA-AC130710 was significantly increased in A375 cells compared with the human primary melanocyte cell line (Figure 3a). We used miRanda and RNAhybrid to predict the putative complementary binding sites of lncRNA-AC130710 and miR-129-5p (Figure 3b).

We further determined whether lncRNA-AC130710 targets miR-129-5p using a luciferase reporter assay. As shown in Figure 3c, overexpression of miR-129-5p decreased the luciferase activity of the wild-type lncRNA-AC130710 3′-UTR reporter in 293T cells. However, miR-129-5p mimics did not influence the luciferase activity of the reporter carrying the mutated lncRNA-AC130710 3′-UTR.

We also constructed lncRNA-AC130710 overexpression plasmids (pcDNA3.1-lncRNA-AC130710) and interference plasmids (lncRNA-AC130710 siRNA) (Figure 3d). The results showed that transfection of pcDNA3.1-lncRNA-AC130710 significantly reduced the level of miR-129-5p; in contrast, transfection of lncRNA-AC130710 siRNA obviously increased the level of miR-129-5p in A375 cells (Figure 3e). We also investigated whether miR-129-5p affected the levels of lncRNA-AC130710. We found that transfection of the miR-129-5p mimic significantly decreased the level of lncRNA-AC130710, indicating that miR-129-5p affected the levels of lncRNA-AC130710 (Figure 3f).

Moreover, modulation of lncRNA-AC130710 expression affected the level of mGluR1 mRNA. As shown in Figure 3g, transfection of pcDNA3.1-lncRNA-AC130710 significantly increased the expression of mGluR1, and lncRNA-AC130710 siRNA obviously decreased the expression of mGluR1 in A375 cells. Subsequently, we investigated the effect of the interaction between lncRNA-AC130710 and miR-129-5p on mGluR1 expression. When cells were transfected with miR-129-5p RNAi, the expression of mGluR1 was increased. Conversely, when cells were transfected with lncRNA-AC130710 RNAi, the expression of mGluR1 was decreased. However, when the levels of lncRNA-AC130710 and miR-129-5p were decreased or both increased, the effect on the expression level of mGluR1 was reversed, i.e., the expression level was almost the same as that in the control group. This indicated that the regulation between lncRNA-AC130710 and mGluR1 might be dependent on miR-129-5p (Figure 3h).

### 3.4 Modulation of lncRNA-AC130710 expression affects cell migration, cell invasion and colony formation in melanoma

**Figure 4:**
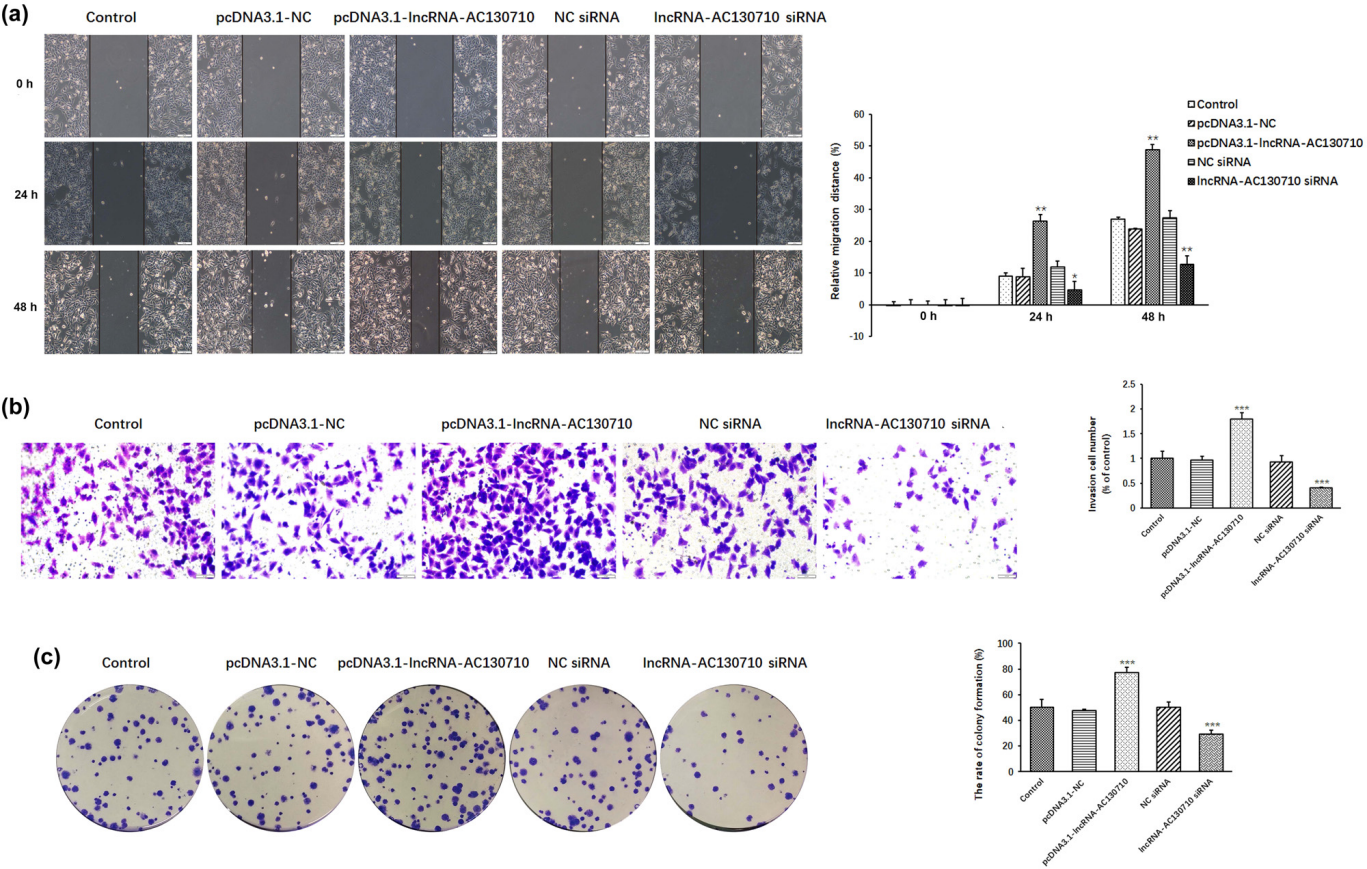
Effects of lncRNA-AC130710 on the migration, invasion and clonal formation of A375 cells. (a) Cell migration was investigated by wound healing scratch assay in A375 cells transfected with pcDNA3.1-lncRNA-AC130710 and lncRNA-AC130710 siRNA vectors. (b) Cell invasion was investigated in A375 cells transfected with pcDNA3.1-lncRNA-AC130710 and lncRNA-AC130710 siRNA vectors. (c) Colony formation of A375 cells transfected with pcDNA3.1-lncRNA-AC130710 and lncRNA-AC130710 siRNA vectors. Data are presented as means ± SD (*n* = 3; *p < 0.05, **p < 0.01, ***p < 0.001).

We examined the effect of lncRNA-AC130710 on cell migration, cell invasion and colony formation in A375 cells (Figure 4). The results of the scratch assay showed that the relative migration distance was 26.29% at 24 h and 48.78% at 48 h in cells transfected with pcDNA3.1-lncRNA-AC130710; however, the relative migration distance was 4.75% at 24 h and 12.66% at 48 h in cells transfected with lncRNA-AC130710 siRNA. The relative migration distance was 8.89% at 24 h and 28.86% at 48 h in control cells (Figure 4a). Overexpression of lncRNA-AC130710 significantly promoted A375 cell migration by 66.15% at 24 h and 44.93% at 48 h compared with the control. Inhibition of lncRNA-AC130710 significantly inhibited A375 cell invasion by 46.64% at 24 h and 52.88% at 48 h compared with the control.

We also examined the effect of lncRNA-AC130710 expression on the invasion of A375 cells (Figure 4b). The results of the invasion assay show that overexpressed lncRNA-AC130710 promotes A375 cell invasion by 80.0%; however, suppressed lncRNA-AC130710 expression inhibits cell invasion by 59.61%.

The effect of lncRNA-AC130710 on the proliferation capacity of tumor cells was also examined with the colony formation assay. pcDNA3.1-lncRNA-AC130710-transfected cells generated a higher rate of colony formation (77.17%) than control cells (50.17%); however, lncRNA-AC130710 siRNA-transfected cells generated a lower rate of colony formation (29.17%) than control cells (Figure 4c).

Overexpression of lncRNA-AC130710 significantly promoted cell invasion, cell migration and colony formation in melanoma, while decreasing lncRNA-AC130710 expression obviously suppressed cell invasion, cell migration and colony formation.

**Figure 5:**
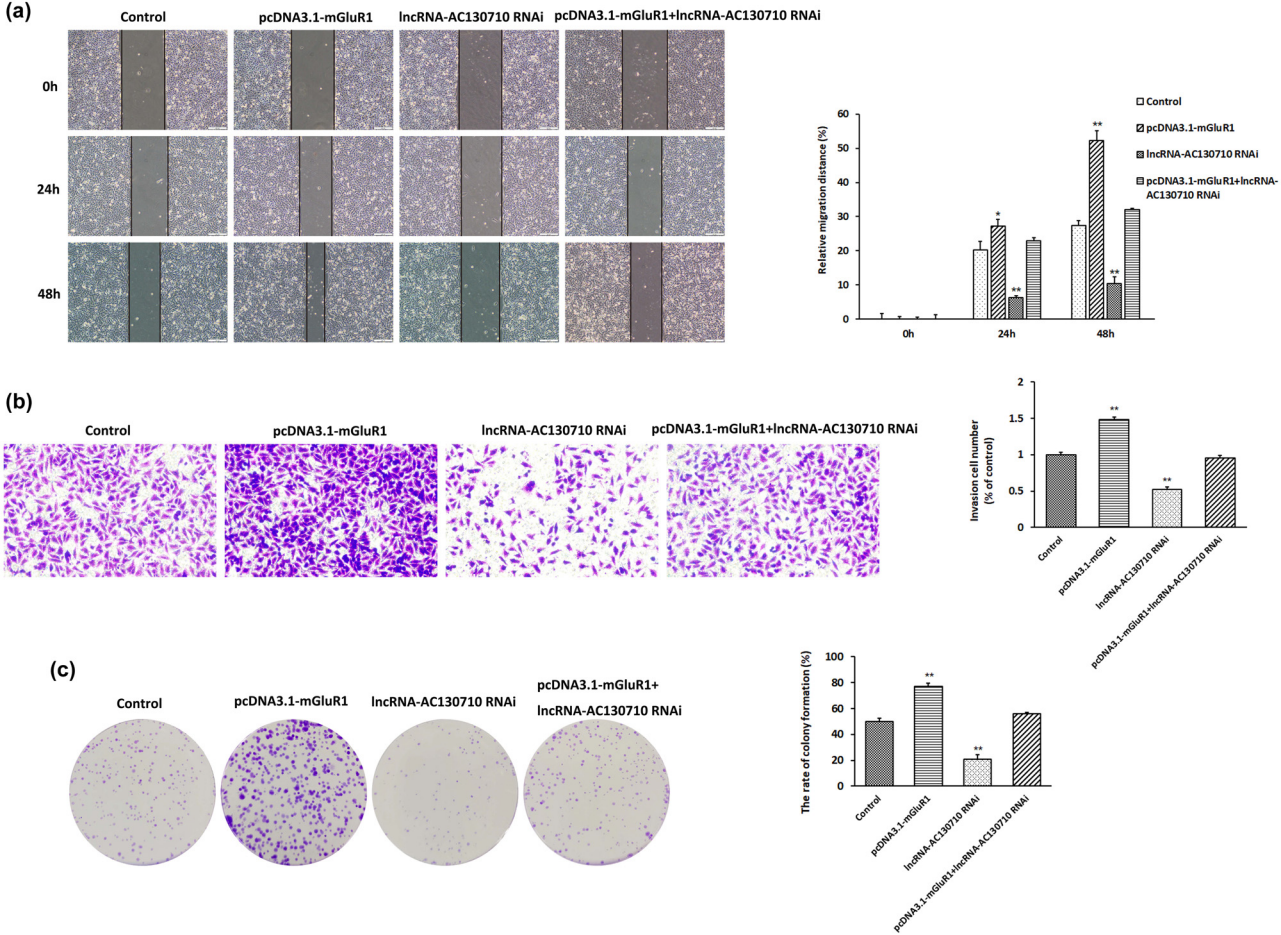
Effects of modulating lncRNA-AC130710 and mGluR1 expression on the migration, invasion and clonal formation of A375 cells. (a) Cell migration was investigated by wound healing scratch assay in A375 cells transfected with pcDNA3.1-mGluR1 and lncRNA-AC130710 siRNA vectors. (b) Cell invasion was investigated in A375 cells transfected with pcDNA3.1-mGluR1 and lncRNA-AC130710 siRNA vectors. (c) Colony formation of A375 cells transfected with pcDNA3.1-mGluR1 and lncRNA-AC130710 siRNA vectors. Data are presented as means ± SD (*n* = 3; *p < 0.05, **p < 0.01).

Furthermore, we wanted to investigate whether the effects of lncRNA-AC130710 expression on cell invasion, cell migration and colony formation depend on mGluR1 levels. We constructed mGluR1 overexpression plasmids (pcDNA3.1-mGluR1) and transfected pcDNA3.1-mGluR1 and lncRNA-AC130710 RNAi into A375 cells. We found that even when mGluR1 was overexpressed, cell invasion, cell migration and colony formation in melanoma were also repressed when lncRNA-AC130710 expression was suppressed in cells (Figure 5a–c).

### 3.5 Expression of miR-129-5p by downregulated lncRNA-AC130710 inactivates the PKCα-MAPK signaling pathway

**Figure 6:**
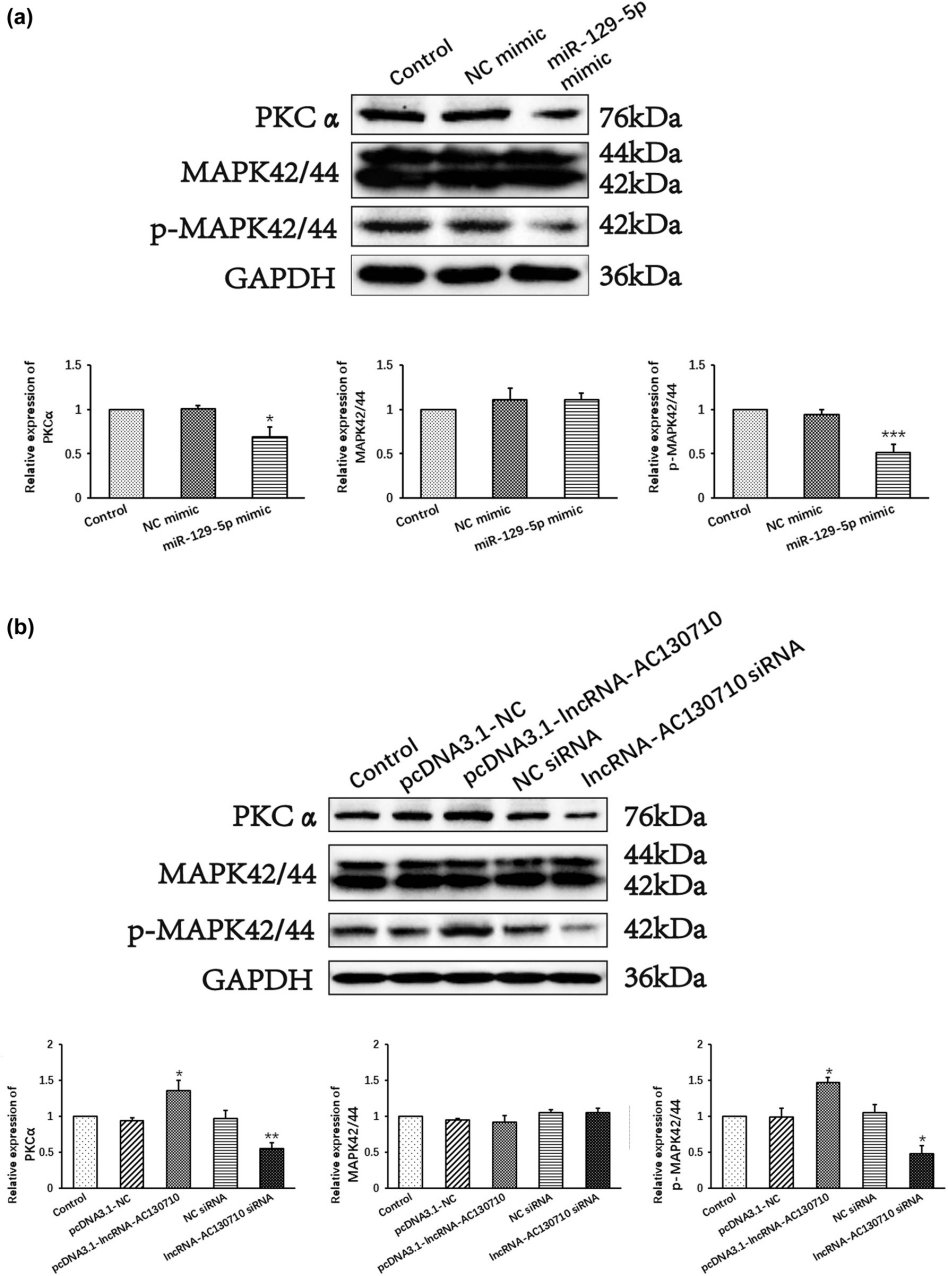
Effect of miR-129-5p and lncRNA-AC130710 on PKCα-MAPK signal pathway. (a) Effect of miR-129-5p overexpression on PKCα-MAPK signal pathway in A375 cells. (b) Effect of lncRNA-AC130710 overexpression and lncRNA-AC130710 silencing on PKCα-MAPK signal pathway in A375 cells. Data are presented as means ± SD (*n* = 3; *p < 0.05, **p < 0.01, ***p < 0.001).

We tested the activation of the PKCα-MAPK pathway to explore the potential underlying mechanisms of the lncRNA-AC130710/miR-129-5p/mGluR1 axis in A375 cells. The results showed that the expression of PKCα and p-MAPK was decreased in miR-129-5p mimic-transfected cells compared with NC mimic-transfected cells (Figure 6a).

Moreover, transfection with pcDNA3.1-lncRNA-AC130710 increased the expression of PKCα and p-MAPK in A375 cells, and transfection with lncRNA-AC130710 siRNA decreased the levels of PKCα and p-MAPK (Figure 6b).

The results indicated that overexpression of miR-129-5p inactivated the PKCα-MAPK pathway. Expression of lncRNA-AC130710 suppressed the regulatory role of miR-129-5p, thereby inactivating the PKCα-MAPK pathway and further promoting cell invasion, cell migration and colony formation in melanoma.

## 4 Discussion

In our study, we found that miR-129-5p suppressed invasion and migration of melanoma cancer cells through targeting mGluR1 transcripts. By suppressing miR-129-5p, lncRNA-AC130710 was capable of promoting metastasis, which was associated with the activation of PKCa-MAPK signal pathway.

In recent years, the glutamate signaling pathway has been reported to be related to tumorigenesis [17]. Glutamate receptors constitute two main groups. One group is comprised of ionotropic receptors, which form ion channels, and the opening and closing of these channels are regulated by glutamate. Methyl-d-aspartate receptor (NMDAR) is a subtype with strong voltage dependence and high Ca^2+^ permeability. The other type is mGluRs, which belongs to the superfamily of G-protein coupled receptors [18]. mGluR1 is a metabotropic glutamate receptor. Lee et al. demonstrated that the expression of mGluR1 in mouse melanocytes was sufficient to induce melanoma [19]. Blocking the activity of GluR1 with an antagonist has been shown to significantly inhibit the invasion and motility of melanoma cells [20]. Previous studies and our results have shown that the expression of mGluR1 is obviously upregulated in melanoma cells [20]. Inhibition of mGluR1 expression can suppress the development of melanoma, including proliferation, cell invasion and cell migration.

Furthermore, we found that miR-129-5p directly targeted and regulated mGluR1. Our results show that miR-129-5p was significantly reduced in melanoma. In addition, the dual luciferase assay verified that miR-129-5p targets mGluR1 and inhibits the expression of mGluR1. Overexpression of miR-129-5p inhibited tumor cell characteristics such as cell migration, cell invasion and clonal formation. Our results demonstrated that inhibiting melanoma can be achieved by regulating miR-129-5p.

LncRNAs function as miRNA sponges to suppress miRNA targeting of mRNAs and the degradation mediated by miRNAs [21,22]. For example, Chen et al. determined that lncRNA FOXD3-AS1 promotes proliferation, invasion and migration of cutaneous malignant melanoma by regulating the miR-325/MAP3K2 axis [23]. Wu et al. indicated that lncRNA MEG3 might inhibit the tumor growth, tumor metastasis and formation of melanoma by modulating the miR-21/E-cadherin axis [24]. Although many studies have described multiple lncRNAs related to melanoma, the involvement of lncRNAs in melanoma tumorigenesis and progression has not been fully studied [25]. A previous study showed that lncRNA-AC130710 directly targets and suppresses miR-129-5p in gastric cancer [16]. LncRNAs target miRNAs through their own miRNA reaction elements at binding sites, further suppressing miRNA targeting of mRNAs and the degradation mediated by miRNAs [26]. In our study, we found that lncRNA-AC130710 has the complementary binding sites miR129-5p, and the dual luciferase experiment also confirmed their relationship. Furthermore, the expression of lnRNA-AC130710 was determined to be significantly increased in melanoma cells. Overexpression of lncRNA-AC130710 reduced the level of miR-129-5p, while downregulation of lncRNA-AC130710 increased the level of miR-129-5p. This result indicated that there is indeed an interactive relationship between them. We also found that overexpression of miR-129-5p significantly decreased the level of lncRNA-AC130710, indicating that miR-129-5p also affected the levels of lncRNA-AC130710. Furthermore, overexpression of lncRNA-AC130710 upregulated mGluR1, while downregulation of lncRNA-AC130710 induced a reduction in mGluR1 expression, demonstrating that the expression of lncRNA-AC130710 suppressed miR-129-5p, a tentative negative regulator of mGluR1 transcripts, and promoted mGluR1 expression increases in melanoma. The regulation between lncRNA-AC130710 and mGluR1 might be dependent on miR-129-5p.

We also demonstrated the effects of modulation of lncRNA-AC130710 expression on cell invasion, cell migration and colony formation in melanoma. We found that overexpression of lncRNA-AC130710 significantly promotes cell invasion, cell migration and colony formation in melanoma, while decreasing lncRNA-AC130710 expression obviously suppresses cell invasion, cell migration and colony formation. Expression of lncRNA-AC130710 suppressed miR-129-5p, a tentative negative regulator of mGluR1 transcripts, which was sufficient to inhibit cell invasion, migration and colony formation in melanoma.

Even after overexpression of mGluR1, cell invasion, cell migration and colony formation in melanoma were also repressed when lncRNA-AC130710 expression was suppressed in cells. These results further demonstrate that miR-129-5p suppressed the migration and invasion of A375 cells by decreasing the expression of mGluR1. However, lncRNA-AC130710 promoted the level of mGluR1 mRNA and the migration and invasion of cells by negatively regulating miR-129-5p.

Activation of NMDAR and mGluR leads to an increase in the activities of PKCα [27]. Inhibiting the expression of mGluR induced the decreased expression or activity of PKCα. The MAPK cascade is considered to be one of the main signaling pathways activated by PKCα [28]. Activation of the MAPK cascade plays a crucial role in many signaling pathways related to cell proliferation. Inhibition of the MAPK cascade can suppress the cell migration and invasion of tumor cells. Our results showed that the expression of lncRNA-AC130710 promoted PKCα activity and MAPK phosphorylation by suppressing miR-129-5p in melanoma, while the downregulation of lncRNA-AC130710 increased miR-129-5p expression and reduced PKCα expression and MAPK phosphorylation. Our study demonstrated that lncRNA-AC130710 promotes cell migration and invasion by suppressing miR-129-5p, which is associated with the activation of the PKCα-MAPK signaling pathway.

## 5 Conclusion

Taken together, our study showed that the lncRNA-AC130710/miR-129-5p/mGluR1 axis plays an important role in the invasion and metastasis of melanoma cell lines.
